# Breeding systems of naturalized versus indigenous species provide support for Baker’s law on Pohnpei island

**DOI:** 10.1093/aobpla/plab038

**Published:** 2021-06-22

**Authors:** Viann Marie Harmony Yomai, Joseph Hill Williams

**Affiliations:** Department of Ecology and Evolutionary Biology, University of Tennessee, Knoxville, TN 37996, USA

**Keywords:** Baker’s law, breeding system, Micronesia, nativity status, Pohnpei, pollen-ovule ratio, reproductive biology, self-compatibility, sexual system

## Abstract

The factors that facilitate successful colonization of islands should be especially evident where the establishment filter is strongest. Colonizers of small, remote oceanic islands should be initially rare, extremely mate-limited and often without pollinators. Hence, plant communities on such islands should reflect an establishment history in which young ‘naturalized’ species are most likely to display self-compatibility and autonomous selfing, whereas ‘indigenous’ species may exhibit more diverse reproductive strategies. To test this prediction, we characterized breeding systems of 28 species on Pohnpei, in the Federated States of Micronesia, a group of remote Pacific islands that are considered a global biodiversity hotspot. Three families with both naturalized and indigenous species were selected—Fabaceae, Malvaceae and Melastomataceae. Measurements included field observations of dichogamy/herkogamy and floral attraction traits, pollen:ovule (*P:O*) ratios and experimental hand-pollinations for self-compatibility and pollen limitation. Phylogenetic generalized least squares analyses tested for trait correlations between naturalized and indigenous species. Flowers of all 28 species were bisexual, and pollinator attraction features were common. Pollen:ovule ratios ranged from 9 to 557 (median = 87), and all 11 hand-pollinated species were self-compatible. All species had >5 ovules and <3500 pollen grains per flower. Indigenous species did not differ significantly from naturalized species for any trait. There is a dearth of data from remote islands bearing on the question of establishment history. In this study, we inferred all species to have some degree of autogamy and indigenous species were no more likely than naturalized species to display outcrossing mechanisms. On Pohnpei, high ovule numbers, and the inaccessibility of wind pollination and obligate outcrossing strategies, reflect the importance of retaining reproductive assurance mechanisms in the face of pollinator uncertainty.

## Teaser

Plants on remote, oceanic islands should reflect an establishment history in which recently ‘naturalized’ colonists can both self-pollinate without pollinators and self-fertilize, whereas older ‘indigenous’ species may evolve outcrossing mechanisms. In the first study of plant breeding systems on Pohnpei island, Micronesia, flowers of 28 species all exhibited overlapping male and female phases and short anther–stigma distances. Low pollen:ovule ratios of 9–557 suggested self-fertilization is common, as revealed by hand self-pollinations of 11 species. Since indigenous were no different than naturalized species, the data support a strong establishment filter and selection for reproductive assurance on oceanic islands.

## Introduction

Oceanic islands have long been considered ‘natural laboratories’ and have provided many key insights into ecological and evolutionary processes (Darwin and Wallace 1858; Barrett 1996). Because island biotas originate largely from the nearest mainland source populations, immigration and extinction rates are dependent on distances to mainland areas and island size, respectively (MacArthur and Wilson 1967). Plant communities on increasingly remote islands should have greater capacity for long-distance dispersal and reproductive traits that allow establishment from single, rather than multiple concurrent, introductions (Baker 1955). Community-level studies of plant reproductive biology have provided insights into colonization history in a number of oceanic island systems (Anderson *et al.* 2001; Lobo *et al.* 2005; Machado *et al.* 2006; Trusty *et al.* 2011; Santos *et al.* 2012), but studies on extremely isolated oceanic islands are still relatively rare (Yorkston 2005; Yorkston and Daehler 2006; Bramwell and Caujape-Castells 2011; Lord 2015).

‘Baker’s law’, as originally stated, indicates that successful establishment of ‘sexually reproducing’ colonies on isolated islands is much more likely to come from a single self-compatible founder than from two self-incompatible or dioecious founders (Baker 1955). The capacity for self-fertilization solves the immediate problems of lack of mates and lack of pollinators, but leads to inbreeding. Hence, Carlquist (1966) argued that the value of outcrossing is so great that many obligate outcrossers in modern oceanic plant communities are likely to have colonized as outcrossers. This led Baker (1967) to point out that ‘Baker’s law’ neither rules out occasional establishment by outcrossers nor the subsequent evolution of outcrossing from self-compatible colonists. Instead, early immigrants ‘are much more likely’ to exhibit traits associated with self-pollination and self-fertilization, whereas longer term persistence must eventually involve shifts to more outcrossed mating systems. Consequently, older immigrants should begin to display modified physical traits that enforce both outcrossing (e.g. dichogamy, dioecy) and cross-pollination (e.g. wind or new local pollinators) (Baker 1967).

To date, tests of Baker’s law have focused on whether or not self-compatibility is over-represented on islands relative to mainlands (Lord 2015; Grossenbacher *et al.* 2017; Razanajatovo *et al.* 2019). To the best of our knowledge, no study has yet explored Baker’s (1967) corollary that there should be historical signal of the establishment filter within modern island plant communities. A comparison of recently naturalized and older, indigenous species might reveal common pathways to successful establishment and persistence. Such an approach has been useful in community studies of invasion biology on mainlands (Burns *et al.* 2011) or large, oceanic islands (references in Schlessman *et al.* 2014). Plant ‘breeding systems’ comprise the morphological and physiological functional traits that determine mating patterns, such as sexual systems, pollination mechanisms, self-incompatibility/compatibility and floral functional morphology (Neal and Anderson 2005). Taken together, such traits can provide insight into evolved pollination and mating patterns.

Under Baker’s scenario, the early stages of establishment typically involve rare or unpredictable pollination environments (Olesen and Jordano 2002) and lack of mates. Thus, both self-pollination and self-compatibility are favoured. Plants in young immigrant populations will have either bisexual flowers and unassisted, intrafloral (autonomous) self-pollination (Crawford *et al.* 2011), or uni- or bisexual flowers and extrafloral (geitonogamous) self-pollination, assisted by gravity, wind or animal pollinators (see Schoen and Lloyd 1992 for different modes of self-pollination).

Later stages of establishment involve accommodation to, or escape from, chronic outcross pollen limitation. The former can occur by shifts to obligate autogamy and reduced inbreeding depression (Pannell *et al.* 2015). The latter, by shifts to new plant–pollinator interactions or to wind pollination (Culley *et al.* 2002; Friedman and Barrett 2009; Lord 2015). Wind pollination is favoured on islands when there are, (i) few animal pollinators (Anderson *et al.* 2001; Schueller 2004; Eckert *et al.* 2006; Kissling and Barrett 2013), or (ii) stronger wind currents (Carta *et al.* 2009), or when, (iii) wind is more effective than animals (Carlquist 1974; Rech *et al.* 2016).

In sum, plant communities on small, isolated islands should be comprised of both recent colonists that predominantly exhibit ‘selfing syndrome’ traits (Sicard and Lenhard 2011) and older indigenous species, that have either evolved stable autogamous mating systems or have transitioned to pollinator-assisted, mixed-mating or even outcrossed systems. Obligate outcrossing is expected to be rare on small, distant islands (for large islands, see Sakai *et al.* 1995; Schlessman *et al.* 2014), since establishment by multiple simultaneous colonists is rare (Baker 1955, 1967), and because small population sizes and high extinction rates (MacArthur and Wilson 1967) limit its evolution. Indigenous or endemic species on such islands should display a greater diversity of reproductive strategies than recent colonists.

 Almost nothing is known about plant reproduction on Micronesian islands (Fosberg and Sachet 1975; Costion and Lorence 2012). The Federated States of Micronesia (FSM) stretches from the mid-Pacific almost to Southeast Asia, in an area just north of the equator. The FSM has been designated a global biodiversity hotspot, since the islands harbour some of the most biologically diverse forests and coral reefs in the world (Wortel 2010). Compared to many Pacific volcanic islands, Pohnpei is relatively old, with volcanic rocks of up to 8.7 million years in age (Rehman *et al.* 2013). Over 1239 species of vascular plants have been described in the FSM. Approximately 782 species are considered native, including 145 species of ferns, 267 species of monocots and 370 species of dicots (Falanruw 2002). More than 200 of these species are endemic to the FSM (Wortel 2010). Islands in the FSM are extremely isolated—for example, the capital island of the FSM, Pohnpei, is 2175 km from the nearest large landmass, Papua New Guinea. However, the majority of the flora and fauna of the FSM is generally thought to have come from the Indo-Pacific region (Indonesia or south-eastern Asia extending out into the Pacific) (Fosberg and Sachet 1975; Keating *et al.* 1984).

Angiosperm breeding systems have not been studied in FSM, and to the best of our knowledge, this is the first study to compare breeding systems in the specific context of establishment history on any oceanic island. The plants of Pohnpei are well-known and the nativity status of each species has been well-documented (Herrera *et al.* 2010). Such knowledge allowed us to test the general prediction that recently arrived, ‘naturalized’ or ‘invasive’ species should be heavily invested in selfing syndrome traits, whereas long-established native ‘indigenous’ or ‘endemic’ species are likely to have evolved more variation in reproductive strategies. We restricted our study to three families of angiosperms that have both naturalized and indigenous species. Our main questions were, (i) what kinds of breeding systems and pollination systems occur on Pohnpei, and (ii) are naturalized species more likely to exhibit breeding system traits that reflect self-pollination and self-fertilization than long-term native species?

## Materials and Methods

### Study site

Pohnpei is the largest (335 km^2^) and highest (~800 m) island in the FSM. Pohnpei has persistent warm, wet conditions year-round, with an average annual temperature of 27.2 °C, and annual precipitation of 5.03 m (Lander and Khosrowpana 2004). The island is divided into five municipalities: Madolenihm (eastern), U (north-eastern), Kitti (southern), Nett (northern) and Sokehs (western).

**Figure 1.**
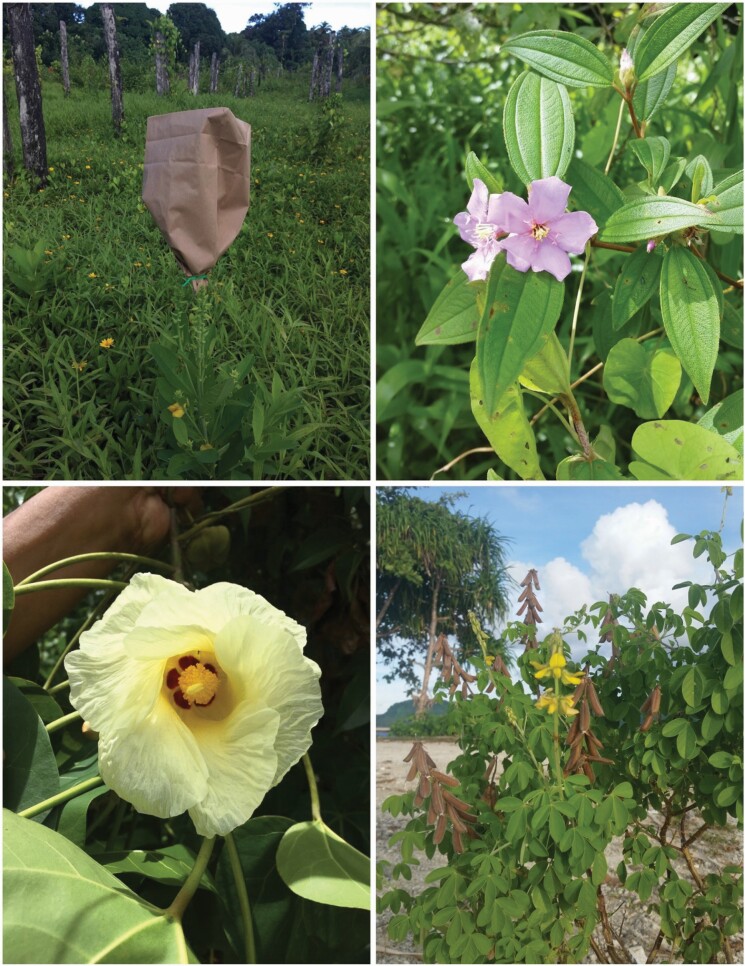
Photos of flowers in the field: (A) bagged flower of *Vigna hosei*, (B) fully open *Melastoma malabathricum* flower, (C) almost fully open *Hibiscus tiliaceus* and (D) *Vigna marina* in the field.

Fabaceae, Malvaceae and Melastomataceae were selected for study (Fig. 1) because each family has both native and naturalized species on Pohnpei. The majority of the flowers were sampled in the understory of forests in Kitti and in coastal areas of Nett. Nativity status (native or naturalized), names and authorities were taken from Herrera *et al.* (2010) (Table 1).

**Table 1.** Species list and authority, nativity status and collection sites. Families arranged order: Melastomataceae, Fabaceae, Malvaceae. Nativity status taken from Herrera *et al.* (2010), and indigenous/endemic species are in shaded rows.

Species	Nativity status	Collection sites	GPS coordinates
*Astronidium ponapense*	Endemic	Forest top	6°54′12″N 158°10′24″E
*Dissotis rotundifolia*	Naturalized	Roadway by stream	6°49′51″N 158°10′28″E
*Melastoma malabathricum*	Indigenous	Open canopy	6°49′51″N 158°10′28″E
*Abrus precatorius*	Naturalized	Understory, roadway	6°49′03″N 158°10′09″E
*Acacia auriculiformis*	Naturalized	Hilltop by road	6°54′26″N 158°09′31″E
*Adenanthera pavonina*	Naturalized	Hilltop by road, abandoned roadway	6°49′08″N 158°14′00″E
*Aeschynome indica*	Naturalized	Open canopy, abandoned land	6°49′11″N 158°11′23″E
*Centrosema molle*	Naturalized	Side of the road	6°53′42″N 158°17′51″E
*Crotalaria pallida*	Naturalized	Open canopy on pepper farm	6°47′52″N 158°14′53″E
*Crotalaria spectabilis*	Naturalized	On the side of the road	6°47′52″N 158°14′53″E
*Dalbergia candenatensis*	Indigenous	On the side of the road	6°49′51″N 158°10′28″E
*Dendrolobium umbellatum*	Indigenous	Roadway by stream, open canopy near stream	6°49′51″N 158°10′28″E
*Derris trifoliata*	Indigenous	Coast, abandoned roadway	6°49′22″N 158°10′06″E
*Leucaena leucocephala*	Naturalized	Roadway by stream, open canopy	6°49′14″N 158°11′30″E
*Mimosa diplotricha*	Invasive	Coast	6°50′27″N 158°09′15″E
*Mimosa pudica* var. *hispida*	Invasive	Coast	6°50′27″N 158°09′15″E
*Senna alata*	Naturalized	Roadside	6°49′11″N 158°11′23″E
*Senna obtusifolia*	Naturalized	Abandoned roadway	6°49′20″N 158°09′54″E
*Senna occidentalis*	Naturalized	Understory	6°47′52″N 158°14′53″E
*Tephrosia candida*	Naturalized	Abandoned roadway	6°49′11″N 158°11′23″E
*Vigna hosei*	Naturalized	Coast	6°58′43″N 158°13′27″E
*Vigna marina*	Indigenous	Coast, beachline	6°58′43″N 158°13′27″E
*Abelmoschus moschatus*	Naturalized	Coast	6°50′27″N 158°09′15″E
*Commersonia bartramia*	Indigenous	By stream, private property	6°50′02″N 158°11′06″E
*Hibiscus tiliaceus*	Indigenous	Forest, roadway	6°48′44″N 158°13′02″E
*Sida acuta*	Invasive	Abandoned roadway, roadside	6°49′13″N 158°11′20″E
*Sida rhombifolia*	Naturalized	Abandoned roadway	6°49′13″N 158°11′20″E
*Thespesia populnea*	Indigenous	Coast	6°50′27″N 158°09′15″E

### Sexual system

Observations of floral sexuality were made in the field. Plant sexual systems were classified as either hermaphroditic (bisexual flowers), monoecious (male and female flowers on the same plant), dioecious (male and female flowers on separate plants) or other rare sexual systems (Tree of Sex Consortium 2014).

### Herkogamy

To determine spatial potential for self-pollination, we measured anther–stigma (A-S) separation. Flowers of each species were first observed at different stages of development, so that herkogamy within each species could be measured at an equivalent stage of sexual maturity. Fabaceae has several flower types that are morphologically distinct: in some species, the keel completely encloses the anthers and stigma, but in others the wings and keel do not completely enclose anthers. For species with complete enclosure by wings and keels, we collected different sizes of flowers in the field, transported them to the lab and dissected them to determine the size at which flowers displayed dehiscing anthers and receptive stigmas. Anther–stigma separation was measured as the minimum distance from the rim of the stigma to either, the top of anthers (if between ovary and stigma), to the bottom of anthers (if further from the ovary than the stigma) or to the middle of the anther if stigmas and anthers were at the same height but not completely touching (Medrano *et al.* 2005). Distances were evaluated in terms of the potential for stigma–anther contact and self-pollination, with positive values indicating stigmas extended significantly further than the anthers, and negative values the converse (Webb and Lloyd 1986; Opedal 2018). Significant A-S separation was determined using 95 % confidence intervals, assuming a normal distribution.

### Dichogamy

The duration of the period separating the presentation of the pollen and receptivity of stigmatic surfaces varies widely between species (Endress 1994). Plants can either have complete dichogamy, where there is no overlap in the presentation of the pollen and stigma receptivity, or incomplete dichogamy, where differences in timing occur, but with some overlap. In protandry, anther dehiscence begins prior to stigma receptivity, and in protogyny, stigma receptivity begins prior to anther dehiscence (Lloyd and Webb 1986; Bertin and Newman 1993).

To determine temporal potential for self-fertilization the timing and duration of floral sexual stages was recorded (closed bud, open flower, anthers undehisced, anthers dehisced and beginning and end of stigma receptivity). Receptivity was determined by observing stigma colour and/or stickiness (Kubitzki and Kurz 1984). Buds of different sizes were dissected to determine at which stage stigmas shifted from being green and dry (non-receptive) to sticky or wet with a translucent, white to neon green colour, which we scored as receptive. Older stigmas withered and became dark yellow to black (non-receptive).

### Pollinator attraction and dispersal traits

Floral colour sometimes reflects the type of pollination vector (Muchhala *et al.* 2014), and we scored colour visually on living flowers (recognizing that insects may perceive colour differently). Floral size can also indicate investment in pollinator attraction and was measured as petal length (banner petal in Fabaceae) on living flowers. Floral reduction can indicate abiotic pollination and/or selfing (Wright *et al.* 2013). Presence or absence of fragrance was determined subjectively by smell on living flowers in the field.

### Pollen:ovule ratios

Pollen:ovule (*P:O*) ratio provides information on breeding system and pollination vector (Cruden 1977). A rough guide, based mostly on mainland species (Cruden 1977), was followed: values < 6 indicate cleistogamy, 6–30 obligate autogamy, 31–180 facultative autogamy, 181–884 facultative outcrossing and >885 obligate outcrossing. Intermediate values were be interpreted in context with other factors, since they are more difficult to interpret in isolation (Cruden 1977; Michalski and Durka 2009). Pollen:ovule ratio was measured using methods from Cruden (1977). Four flower buds per species for members of all three families were sampled in the field and stored in 70 % ethanol. In the lab, four anthers per bud were counted for Fabaceae and Melastomataceae and 10 for Malvaceae. The 10 anthers for Malvaceae were sampled evenly from the bottom-most to the top-most anther. All anthers were placed on microscope slides and crushed with cover slips. Some Malvaceae pollen grains were clumped together, so detergent was used to separate clumps. From there, a subsample of known volume of the pollen–detergent mixture was placed on microscope slides and pollen grains were counted under the microscope. Pollen grain number per flower was calculated by scaling to the total number of anthers or subsamples per anther for each flower. Ovaries were dissected and ovules were hand-counted [see Supporting Information—File 4]. Pollen diameter was measured along the longest equatorial axis on pollen preserved in fuschin gel [see Supporting Information—File 3] (Pacini and Hesse 2005).

### Experimental hand-pollinations

Eleven species were selected for hand-pollination experiments to test for self-compatibility and pollen limitation. Four flowers on each of *N* = 5 plants were treated as follows: (i) *Hand-self*: to measure the degree of self-compatibility, pollen grains were deposited on stigmas of the same flower and bagged to exclude pollinators (Fig. 1). (ii) *Autonomous-self*: to measure unassisted, within-flower self-pollination success, flowers were bagged prior to anther dehiscence and stigma receptivity to completely exclude pollinators. (iii) *Open-pollination*: to measure extrafloral pollination success (by geitonogamy or outcrossing), flowers were emasculated prior to anther dehiscence and left unbagged for pollinators to visit. (iv) *Hand-outcross*: to measure outcross pollination success, flowers were emasculated and stigmas were rubbed with open anthers of flowers from different plants. All treatments were visited 2–3 weeks later and developing seeds were counted. Seed set was scored as the number of developing seeds per flower [see Supporting Information—File 5].

A number of breeding system indices were calculated from relative seed set of the experimental treatments, as shown in Box 1. For ISI, negative values were set to zero. For OCI, flower width was doubled petal length for Malvaceae and Melastomataceae and wing petal length for Fabaceae. ‘Success’ in self- versus outcross comparisons was measured as seed set. Note that *OCI*, *ISI*, and *P:O* ratio range from zero (more autogamous) to one (more outcrossing), whereas AI and AF are the reverse (Box 1). Pollen limitation (*PL*) can be measured a number of different ways (Box 1). Our *PL* indices measured *pollination vector-assisted pollen limitation*, since the open-pollination treatment excluded autonomous self-pollination but allowed both geitonogamy and outcrossing. Thus, lower open seed set than either or both outcrossed or selfed seed set indicates pollen limitation due to lack of a pollination vector.

Box 1. **Breeding system indices. S, self; OC, outcross.**

**Table:** 

Abbreviation	Formulas and rationale	References
*OCI*	*Floral outcrossing index* *OCI* = sum of scores below *Range*: 0 (cleistogamy) to 5 (partially self-compatible, requires pollinators)	Cruden (1977); Dafni (1992)
	Diameter of floral ‘target’ (flower or head): <1 mm = 0, 1–2 mm = 1, 2–6 mm = 2, >6 mm = 3	
	Herkogamy: absent = 0, reverse or approach herkogamy = 1* *consider the degree of anther–stigma separation that prevents selfing	Webb and Lloyd (1986); Opedal (2018)
	Dichogamy: absent, overlapping or protogyny = 0, protandry = 1	Lloyd and Webb (1986)
	*Strength*: rough field-measure of floral attractiveness to pollinator-assisted outcrossing *Weakness*: does not preclude pollinator-assisted selfing among flowers (geitonogamy)	
Sexual system	*Sexual system* Description of distribution of floral sex organs All flowers on plant cosexual (hermaphrodite); flowers unisexual and both sexes on same plant (monoecy), or each sex on separate plant (dioecy); and eight other combinations or variants	Tree of Sex Consortium (2014); Lord (2015)
*ISI*	*Index of self-incompatibility* ISI = 1 − (hand-self success/hand-outcross success^A^)	Lloyd (1965)
	*Range*: ≤0.2 autogamy; ≤0.8 self-compatible; and >0.8 highly outcrossing	Raduski *et al.* (2012)
	*Strength*: rough measure of self-sterility; correlated with mating system *Weakness*: does not indicate mechanism of self-sterility; cut-off values are arbitrary; values < 0 can indicate outbreeding depression, outcrossing with relatives, handling errors or small sample size	Gibbs (2014); Raduski *et al.* (2012)
*AI*	*Autogamy index* *AI* = autonomous self-success/hand-outcross-success^A^ *Range*: 0 (low) to >1 (high)	Ramirez and Brito (1990)
	*Strength*: high values suggest autogamous mating system, especially if *AF*, below, is also high *Weakness*: apomixis can inflate value; high values can also indicate handling problems	Cavalcante *et al.* (2020)
*AF*	*Autofertility index* *AF* = autonomous-self-success/hand-self-success^A^ *Range*: 0 (low) to 1 (high) autofertility (values > 1, high autofertility, but handling errors or imperfect timing of hand-pollination)	Schoen and Lloyd (1992)
	*Strength*: measures magnitude of unassisted, intrafloral self-fertilization (by unassisted self-pollination) relative to maximum possible *Weakness*: only useful if substantial self-success is found; apomixis not excluded	
	*Alternative tests for mechanisms of selfing*:	See Lloyd (1992); Schoen and Lloyd (1992)
*P:O ratio*	*Pollen to ovule ratio* *P:O* ratio = total pollen grains to total ovules per flower Range: values near zero indicate selfing and/or stable pollination ecology, higher values outcrossing and/or inefficient pollination	Cruden (1977)
	*Strength*: measures relative allocation to male and female function per flower, an evolved response to long-term pollination efficiency; simple to measure and roughly correlates with degree of selfing or outcrossing, especially if controls are considered (taxonomic group, ecology, etc.) *Weakness*: pollination environment and degree of outcrossing often co-vary; intermediate values can be difficult to interpret	Michalski and Durka (2009)
*PL*	*Pollen limitation index* (floral level) *PL* = 1 − (open-pollination/supplemental pollination success^A^) Range: 0 (no pollen limitation) to 1 (severe pollen limitation); values < 0 can indicate handling or over-pollination effects, or statistical error *Alternatives for numerator/denominator* (*bolded most common*). General *PL* (1, 3 OC); this study (2, 5 S or OC − pollinator-assisted *PL*) ^**1**^**Open: no emasculation = autonomous (a) + geitonogamous (g) + OC pollinations allowed** ^2^Open: emasculation = g + OC (only assisted open-pollination allowed) ^**3**^**Suppl: no emasculation/bagging = a + g + OC allowed; S or OC applied** ^4^Suppl: emasculation, no bagging = g + OC allowed; S or OC applied ^5^Suppl: emasculation, bag = only S or OC hand-pollination allowed	Larson and Barrett (2000)
	*Strength*: measures the degree to which seed set is limited by self or outcross pollen reception at the floral level (whole plant level more meaningful) *Weakness*: subject to variation in pollination environment, resource re-allocation among flowers within the plant and within and among years; confounds actual pollination limitation with siring success	Burd (1994); Knight *et al.* (2005); Burns *et al.* (2019)
	*Alternative*: actual per-flower natural stigmatic pollen load-to-ovule number	Snow (1986)

^A^‘Success’ can be measured at different stages, such as by differences in stigmatic pollen loads, pollen tube numbers or lengths, fruit set, ovule set or seed set.

### Statistical analyses

All statistical analyses were conducted in R version 3.6.2 (R Core Team 2020). We generated a phylogenetic tree by extracting species from Smith and Brown (2018) using the V.Phylo.Maker package in R with a single tip per species (Jin and Qian 2019). For each continuous trait, we tested for differences between recent colonists (species categorized as ‘invasive’ or ‘naturalized’) versus ancient colonists (‘indigenous’ or ‘endemic’ species) (as in Herrera *et al.* 2010). Values were log_10_-transformed for analysis. We used nativity status as a categorical predictor variable in phylogenetic generalized least squares (PGLS) under various models of evolution and in non-phylogenetic generalized least squares (GLS) in APE (Paradis *et al.* 2004). Models with lowest AIC_C_ were retained. Phylogenetic signal was assessed using Pagel’s *λ* (Pagel 1999). To compare experimental pollinations, separate ANOVAs were done for each species, with seed number per flower as the response variable. Means were separated using Tukey’s HSD test [see Supporting Information—File 1].

## Results

### Floral traits related to pollinator attraction and sexual function

All species had bisexual flowers. No floral trait was associated with nativity status. Floral sizes varied by family (Table 2), with petal length ranging from 0.5 to 29 mm in Fabaceae, and 8 to 108 mm in Malvaceae. Naturalized Melastomataceae species (*Dissotis rotundifolia* and *Melastoma malabathricum*) had longer petals than the endemic, *Astronidium ponapense,* but the difference was not significant. Petal length was not associated with nativity status (*λ* = 1.01; *N* = 17, 8; *P* = 0.743; see Supporting Information—File 2).

**Table 2.** Floral traits related to pollination system. All values are means of three flowers per species (±SD for petal length). Herkogamy is A-S separation, positive if stigma height > anther height, negative if the reverse (±½ 95 % confidence interval). Dichogamy was either incomplete protandry (IPDR) or incomplete protogyny (IPGY). For fragrance, D indicates fragrance detected, and N indicates fragrance not detected (Dafni 1992). Families arranged in order: Melastomataceae, Fabaceae, Malvaceae. Shaded rows indicate native species.

Species	Petal length (mm)	Herkogamy (mm)	Dichogamy	Fragrance	Colour	*OCI* index
*Astronidium ponapense* ^E^	7 ± 0.3	−0.47 ± 0.02	IPGY	N	Maroon-red	4
*Dissotis rotundifolia* ^N^	14 ± 0	−0.31 ± 0.02	IPGY	N	Purple	4
*Melastoma malabathricum* ^I^	14.7 ± 0.2	−0.27 ± 0.01	IPGY	N	Purple	4
*Abrus precatorius* ^N^	0.5 ± 0.09	−2.98 ± 0.39	IPGY	N	Red	1
*Acacia auriculiformis* ^N^	0.7 ± 0.8	1.00 ± 0.00		D	Yellow	1
*Adenanthera pavonina* ^N^	1.7 ± 0.6	0	IPGY	D	White	1
*Aeschynome indica* ^N^	7 ± 0	0.1 ± 0.05	IPGY	N	Yellow	4
*Centrosema molle* ^N^	29 ± 1.9	−0.3 ± 0.02	IPGY	N	Purple	4
*Crotalaria pallida* ^N^	12 ± 0.8	0.2 ± 0.04	IPGY	N	Yellow	4
*Crotalaria spectabilis* ^N^	11.3 ± 1	0.3 ± 0.05	IPGY	N	Yellow	4
*Dalbergia candenatensis* ^I^	8 ± 1.2	0.5 ± 0.03	IPGY	N	Light purple	4
*Dendrolobium umbellatum* ^I^	4 ± 0.7	1 ± 0.05	IPGY	N	White (purple)	3
*Derris trifoliata* ^I^	7 ± 0.9	1.00 ± 0.00	IPGY	N	White	4
*Leucaena leucocephala* ^N^	17 ± 0.2	1.00 ± 0.00	IPGY	D	White (off)	4
*Mimosa diplotricha* ^INV^	11.7 ± 2	−3 ± 0.22	IPGY	N	Purple	4
*Mimosa pudica* ^INV^	13 ± 1.3	−2 ± 0.08	IPGY	N	Purple	4
*Senna alata* ^N^	17 ± 2.1	1 ± 1	IPGY	N	Yellow	3
*Senna obtusifolia* ^N^	23 ± 1.5	−0.5 ± 0.13	IPGY	N	Light yellow	4
*Senna occidentalis* ^N^	16.4 ± 0.3	0	IPGY	N	Yellow (orange)	3
*Tephrosia candida* ^N^	10 ± 0	−0.7 ± 0.10	IPGY	N	White	4
*Vigna hosei* ^N^	13 ± 0.1	2.3 ± 0.14	IPGY	N	Yellow	4
*Vigna marina* ^I^	12 ± 0.1	2 ± 0.05	IPGY	N	Yellow	4
*Abelmoschus moschatus* ^N^	108 ± 2.3	8 ± 0.55^a^	IPDR	D	Light green	4
*Commersonia bartramia* ^I^	13 ± 0.6	−1 ± 0.04	IPDR	D	White	4
*Hibiscus tiliaceus* ^I^	76 ± 0.07	2 ± 0.59^a^	IPDR	N	Light green	4
*Sida acuta* ^INV^	8 ± 0.02	1 ± 0.18	IPDR	N	Yellow	4
*Sida rhombifolia* ^N^	6.3 ± 0.34	0.5 ± 0.3	IPDR	N	Yellow	4
*Thespesia populnea* ^I^	100 ± 2.1	11 ± 1.41^a^	IPDR	N	Off white	4

^a^Anthers were same height as stigma but separation distance was lateral.

^E^Endemic,

^I^Indigenous,

^N^Naturalized and

^I^ ^N^ ^V^Invasive.

Floral colours tended to be bright—only two species had a non-showy (green) flower colour (Table 2)—however, fragrance was not only detected in three Fabaceae species (*Acacia auriculiformis, Adenanthera pavonia, Leucaena leucocephala*) and two Malvaceae species (*Abelmoschus moschatus* and *Commersonia bartramia*). Although we did not formally search for pollinators, during collection, only ants, grasshoppers and caterpillars were seen on the flowers, and these are classes of insects not generally known to be pollinators.

All flowers were more or less upright, and all but two species (Fabaceae) had significant A-S separation, or herkogamy. Most Fabaceae and Malvaceae had positive herkogamy (anthers positioned between ovary and stigma), whereas all Melastomataceae, one Malvaceae and six Fabaceae had anthers extended beyond the stigma (Table 2). All species exhibited incomplete dichogamy: incomplete protogyny in all Melastomataceae and Fabaceae, and incomplete protandry in all Malvaceae (Table 2).

All three Melastomataceae had high *OCI* scores due to their wide flowers and significant, although slight, A-S separation (<0.5 mm in all) (Table 2). In Fabaceae, two species had very small petals (<1 mm, +0 points), two species were intermediate (+1 or 2 points) and the rest were >6 mm (+3 points). All but three scored +1 for herkogamy, but herkogamy was ≤0.5 mm in six of these, and all scored +0 for their partially overlapping female and male sexual phases. Malvaceae had the highest *OCI* scores due to their generally large flowers and strong herkogamy (all +4), though all had incomplete protandry.

### *P:O* ratios

Pollen to ovule ratios across all species ranged from 8 to 557, with a median of 86 (Table 3). In Melastomataceae, flowers had c. 1600–2000 pollen grains of small size (<25 µm diameter) with many ovules (>100), resulting in very low *P:O* ratios (median *P:O* ratio = 16.4). On the other hand, Malvaceae flowers generally produced fewer numbers (80–2200) of very large pollen grains (>82 µm diameter) and fewer ovules, resulting in *P:O* ratios that ranged from 9 to 159 (median = 13.6). Fabaceae had small to medium-sized pollen grains of variable number (538–3344) and few ovules, and generally had much higher *P:O* ratios, ranging from 20 up to 557 (median 131.0). Pollen:ovule ratio was not significantly different between naturalized and indigenous species (*λ* = 0.543; *N* = 23; *P* = 0.922).

**Table 3.** Pollen and ovule numbers and *P:O* ratio. Pollen grains are estimated number of pollen grains from four anthers per flower in Fabaceae and Melastomataceae and 10 anthers per flower in Malvaceae. Ovule numbers taken from the same four flowers from which anthers were collected. Values are means (±1 SD). Shaded rows indicate native species.

Species	Pollen size (µm)	Pollen grains	Ovules	*P:O* ratio
*Astronidium ponapense* ^E^	24 ± 1.0	1993.1 ± 12.6	100 ± 3.8	19.93 ± 0.48
*Dissotis rotundifolia* ^N^	23 ± 0.0	1605.3 ± 48.7	103 ± 3.5	15.6 ± 0.6
*Melastoma malabathricum* ^I^	20 ± 0.2	1670.0 ± 80.8	101.8 ± 4.1	16.4 ± 0.4
*Abrus precatorius* ^N^	41 ± 0.0	1388.3 ± 36.9	8.0 ± 0	173.5 ± 1.7
*Acacia auriculiformis* ^N^	38 ± 0.2	1292.5 ± 45.0	26.5 ± 4.0	49.4 ± 4.9
*Adenanthera pavonina* ^N^	41 ± 3.8	1653.9 ± 17.3	25.0 ± 0.0	66.2 ± 0.2
*Aeschynome indica* ^N^	25 ± 0.0	1508.7 ± 35.7	8.0 ± 0.0	188.6 ± 2.4
*Centrosema molle* ^N^	32 ± 6.2	1322.8 ± 22.3	20.0 ± 0.0	66.1 ± 0.9
*Crotalaria pallida* ^N^	42 ± 0.1	1394.8 ± 32.3	16.0 ± 2.0	87.2 ± 1.1
*Crotalaria spectabilis* ^N^	27 ± 1.0	1500.8 ± 31.4	10.0 ± 0.0	150.1 ± 3.2
*Dalbergia candenatensis* ^I^	14 ± 0.0	1830.3 ± 23.1	25.0 ± 0.0	73.2 ± 0.2
*Dendrolobium umbellatum* ^I^	27 ± 0.2	1788.1 ± 54.1	6.0 ± 0.0	298.0 ± 8.2
*Derris trifoliata* ^I^	13 ± 0.8	3343.3 ± 278.0	6.0 ± 0.0	557.2 ± 35.9
*Senna alata* ^N^	31 ± 3.0	NA	19.3 ± 3.1	NA
*Senna obtusifolia* ^N^	25 ± 3.8	538.0 ± 17.4	26.8 ± 2.4	20.2 ± 1.4
*Senna occidentalis* ^N^	28 ± 4.8	2500.0 ± 48.7	29.0 ± 1.4	86.3 ± 0.4
*Tephrosia candida* ^N^	NA	1342.3 ± 33.3	10.0 ± 0.0	134.2 ± 1.0
*Vigna hosei* ^N^	17 ± 2.6	1331.6 ± 47.0	6.0 ± 0.0	222.0 ± 6.0
*Vigna marina* ^I^	20 ± 3.0	1298.1 ± 50.8	6.0 ± 0.0	216.4 ± 1.0
*Abelmoschus moschatus* ^N^	163 ± 6.4	NA	142.3 ± 0.81	NA
*Commersonia bartramia* ^I^	94 ± 8.6	NA	25.0 ± 0.0	NA
*Hibiscus tiliaceus* ^I^	173 ± 5.0	NA	23.3 ± 2.7	NA
*Sida acuta* ^INV^	83 ± 0.8	NA	10.0 ± 0.0	NA
*Sida rhombifolia* ^N^	128 ± 0.4	1587.8 ± 69.7	10.0 ± 0.0	158.8 ± 2.6
*Thespesia populnea* ^I^	172 ± 0.2	NA	25 ± 3.21	NA

^E^Endemic,

^I^Indigenous,

^N^Naturalized and

^I^ ^N^ ^V^Invasive.

Ovule numbers did not differ between naturalized and indigenous species (*λ* = 1.290; *N* = 23; *P* = 0.648). Pollen size and pollen number also showed no significant difference for nativity status (for size, *λ* = 0.608; *P* = 0.2713; for number, *λ* = 0.504; *P* = 0.377; both *N* = 23). There was a significant negative correlation between pollen size and pollen number (log–log slope = −0.971; *λ* = 0.503; *N* = 23; *P* = 0.001), but that result that was not affected by nativity status (*λ* = 0.055; *N =* 15, 8; *P* = 0.226).

### Hand-cross pollinations

Within all 11 species, the hand-self crosses produced a higher or similar number of seeds as the hand-outcrosses (Table 4). In addition, all but one species produced seeds under autonomous self-pollination. Six open-pollinated treatments produced more seeds than hand-outcross treatments, a result that could be not be due to autonomous selfing but may include geitonogamous pollinations (Table 4). Open-pollination seed numbers were lower than either one or both of the hand-self or outcross treatments, an indicator of self or outcross pollen limitation. All species were self-compatible, with *ISI* < 0.8, and all but two Fabaceae had *AF* values < 0.2.

**Table 4.** Hand-pollination experiments. Values represent the average number of seeds per *N =* 5 flowers (1 SE). In each row, values with different superscripts are significantly different. ‘Hand-self’, hand-self pollinations; ‘Autonomous-self’, bagged and unmanipulated; ‘Open-pollination’, emasculated without bagging; ‘Hand-outcross’, emasculated, cross-pollinated. Shaded rows indicate native species.

Species	Hand-self	Autonomous-self	Open-pollination	Hand-outcross
*Crotalaria pallida* ^N^	20.6 (1.78)^A^	18.8 (2.06)^A^	5.2 (2.33)^B^	1.2 (1.2)^B^
*Dendrolobium umbellatum* ^I^	3 (0.32)^A^	2.6 (0.51)^A^	0.2 (0.2)^B^	0.6 (0.4)^B^
*Leucaena leucocephala* ^N^	13.6 (1.72)^A^	11.4 (1.17)^A^	6 (3.29)^AB^	0.8 (0.8)^B^
*Mimosa diplotricha* ^INV^	7.6 (0.4)^A^	0.4 (0.4)^B^	6.8 (0.8)^A^	0 (0)^B^
*Mimosa pudica* ^INV^	11.2 (1.62)^A^	0 (0)^B^	9.2 (0.8)^A^	0 (0)^B^
*Senna alata* ^N^	5.6 (0.4)^A^	6.2 (0.66)^A^	4 (1.05)^A^	6 (0.32)^A^
*Vigna marina* ^I^	4.6 (0.24)^A^	4 (0.55)^A^	1 (0.32)^B^	3.8 (0.49)^A^
*Hibiscus tiliaceus* ^I^	53 (1.05)^A^	52.2 (0.86)^A^	17.4 (2.54)^B^	47.8 (0.86)^A^
*Sida acuta* ^INV^	51.2 (2.33)^A^	24.4 (5.68)^B^	40.4 (5.17)^AB^	52.6 (3.03)^A^
*Sida rhombifolia* ^N^	3 (0.45)^A^	6.8 (0.8)^B^	6.4 (0.4)^B^	1.4 (0.68)^A^
*Thespesia populnea* ^I^	14.2 (0.8)^A^	5.8 (1.07)^B^	13 (2)^A^	11.2 (0.58)^A^

^E^Endemic,

^I^Indigenous,

^N^Naturalized and

^I^ ^N^ ^V^Invasive.

## Discussion

In this study, we addressed the corollary to Baker’s law (Baker 1967)—if establishment on islands is more likely by self-pollinating, self-compatible species, then cross-pollination and outcrossing mechanisms in island plant communities should generally have evolved from self-fertilizing founders. The best cases for such a scenario should be found on remote oceanic islands. Thus, we tested the prediction that recently established species on Pohnpei would have high levels of selfing syndrome traits, whereas indigenous species might exhibit more diverse reproductive strategies. Instead, we found universal self-compatibility in self-crosses, and minimal differences between naturalized and indigenous species in breeding system traits. Almost all species had low *P:O* ratios and floral morphologies consistent with selfing syndromes (Cruden 1977; Sicard and Lenhard 2011; Cutter 2019). Autonomous self-pollination was also common, suggesting that the low *P:O* ratios reflect a general reliance on self-fertilization. We conclude that all 28 species have some degree of autogamy in their breeding system, which likely reflects strong selection for autogamy during early establishment and limited pathways to evolving highly outcrossed mating systems.

### Floral traits as indicator of pollination systems of Pohnpei

A majority of the species studied had some pollinator attraction features, such as showy flowers with large petals and some had fragrance. Floral phenotypes may not perfectly predict specific pollinators of most plant species (Ollerton *et al.* 2015), but there are broad and consistent sets of traits that differentiate outcrossed animal-, wind- and self-pollinated flowers. Animal-pollinated flowers usually display bright colours, large showy petals, and strong fragrance or pollen rewards that attract potential pollinators such as bats, birds and insects (Stroo 2000; Kessler *et al.* 2008; Muchhala *et al.* 2014; Edger *et al.* 2015). Abiotic pollinated flowers are almost the exact opposite: non-showy flowers, no fragrance and small petals (Culley *et al.* 2002; Friedman and Barrett 2009), but with many pollen grains. Habitually self-pollinated flowers are also typically less attractive and smaller (Hetherington-Rauth and Johnson 2020), but with few pollen grains, incomplete dichogamy and lack of herkogamy.

There were few signs of wind pollination on Pohnpei. In Malvaceae, pollen grains were 83–163 µm diameter, which is much larger than the 17–58 µm range seen in most wind-pollinated species (Friedman and Barrett 2009). In Melastomataceae and Fabaceae, pollen production was strikingly low and no species had fewer than six ovules per flower. Wind-pollinated species tend to produce high amounts of pollen, and one or only a few ovules per flower (Friedman and Barrett 2009). Furthermore, anthers of Melastomataceae are poricidal, which is typical of animal pollination. Finally, species with the highest *P:O* ratios were inconsistent with wind pollination—they had either high floral attraction scores (e.g. *Aeschynome*, *Derris*, *Vigna*), or strong indicators of obligate autogamy (*Abrus*), or both (*Dendrolobium*, *Hibiscus tiliaceous*, *Sida rhombifolia*, *Vigna marina*) (Table 5). Thus, the incomplete and variable retention of features like showy petals, large pollen grains and floral fragrance in a few species suggests either occasional animal pollination or the retention of ancestral outcrossing characters.

**Table 5.** Summary of breeding indices and inferred breeding systems. If numerator and denominator in an index were not significantly different, then ‘~1’ or ‘~0’, otherwise values shown. *ISI*, index of self-incompatibility; *AI*, autogamy index; *AF*, autofertility index; *PL*, pollen limitation index—if seed set of open (geitonogamous or outcross) pollination was lower than either outcross or hand-self seed set, ‘PL’, or if non-significant ‘~0’ (no *PL*); *OCI*, outcrossing index; *P:O* ratio, pollen to ovule ratio. See text for discussion of inferred breeding systems: FA, facultative autogamy; FO, facultative outcrossing; SC, species with self-compatibility based on crossing data. ‘Mixed’ indicates either FA or FO. Shaded rows indicate native species.

Species	*ISI*	*AI*	*AF*	*PL*	*OCI*	*P:O* ratio	Inferred breeding system
*Astronodium ponapense* ^E^					4	20	Autogamous
*Dissotis rotundifolia* ^N^					4	16	Autogamous
*Melastoma malabathricum* ^I^					4	16	Autogamous
*Abrus precatorius* ^N^					1	174	FA
*Acacia auriculiformis* ^N^					1	50	FA
*Adenanthera pavonia* ^N^					1	66	FA
*Aeschynome indica* ^N^					4	189	Mixed
*Centrosema molle* ^N^					4	66	FA
*Crotalaria pallida* ^N^	<0	>1	~1	PL	4	87	SC: FA
*Crotalaria spectabilis* ^N^					4	150	FA
*Dalbergia candenatensis* ^I^					4	73	FA
*Dendrolobium umbellatum* ^I^	<0	>1	~1	PL	3	298	SC: Mixed
*Derris trifoliata* ^I^					4	557	FO
*Leucaena leucocephala* ^N^	<0	>1	~1	~0	4		SC: FA
*Mimosa diplotricha* ^INV^	NA	NA	0.05	PL	4		SC: FA
*Mimosa pudica* ^INV^	NA	NA	0	PL	4		SC: FA
*Senna alata* ^N^	~0	~1	~1	~0	3	51	SC: FA
*Senna obtusifolia* ^N^					4	20	Autogamous
*Senna occidentalis* ^N^					3	86	FA
*Tephrosia candida* ^N^					4	134	FA
*Vigna hosei* ^N^					4	222	Mixed
*Vigna marina* ^N^	~0	~1	~1	PL	4	217	SC: Mixed
*Abelmoschus moschatus* ^N^					4	17	Autogamous
*Commersonia bartramia* ^I^					4	9	Autogamous
*Hibiscus tiliaceus* ^I^	<0	>1	~1	~0	4	107	SC: FA
*Sida acuta* ^INV^	~0	0.46	0.48	~0	4	8	SC: Autogamous
*Sida rhombifolia* ^N^	~0	>1	>1	~0	4	159	SC: FA
*Thespesia populnea* ^I^	~0	0.52	0.41	~0	4	10	SC: Autogamous

^E^Endemic,

^I^Indigenous,

^N^Naturalized and

^I^ ^N^ ^V^Invasive.

### Floral traits as indicators of breeding systems of plants of Pohnpei

Flowers of all species were bisexual, which allows the potential for intrafloral, autonomous self-pollination. Melastomataceae and Fabaceae flowers had incomplete protogyny, which is usually associated with outcrossing species that have delayed selfing (Webb and Lloyd 1986; Goodwillie and Weber 2018), a good mechanism for reproductive assurance, since extrafloral pollination can occur prior to intrafloral selfing. All Malvaceae had incomplete protandry. Protandry is much more common than protogyny (Bertin and Newman 1993) and is thought to be a better solution to the problem of interference from self-pollen on the stigma when outcrossing is common (Lloyd *et al.* 1980; Lloyd and Webb 1986). In incomplete protandry the onset of stigma receptivity occurs before the pollen presentation period has ended. However, some Malvaceae species had reproductive assurance via delayed selfing despite incomplete protrandry, since the stigmas eventually curved over and touched still dehiscent anthers (*Sida acuta* and *S. rhombifolia*).

Flowers of all Melastomataceae and six (of 19 species) Fabaceae exhibited ‘reverse herkogamy’ in which a pollinator would contact the anther before the stigma. The converse, ‘approach herkogamy’ was more common, and is often seen in self-compatible species with more specialized pollination systems (Opedal 2018). Most species had low herkogamy: A-S distance was ≤ 1 mm in 18 species, whereas only two Malvids had A-S separation ≥ 3 mm. Given the partial overlap between anther dehiscence and stigma receptivity in all species, these results suggest substantial potential for selfing.

### Pollination efficiency based on *P:O* ratios

Pollen to ovule ratios are a good indicator of long-term pollination conditions—extremely low *P:O* ratios reflect highly efficient pollination systems such as in autogamous systems, whereas extremely high *P:O* ratios are typical of more inefficient systems such as with high outcrossing, wind pollination or unpredictable pollination environments (Cruden 1977). Based on *P:O* ratio alone, all of our species were fully or partially autogamous (Table 5). Values in all three Melastomes are consistent with cleistogamy (*P:O* ratio < 30), and both cleistogamous and chasmogamous flowers were seen in all three species in the field. A survey of neotropical mainland Melastomataceae by Santos *et al.* (2012) found 80 % of species were either self-compatible or apomictic. We did not distinguish apomixis from cleistogamy (Uphof 1938); however, the high number of ovules (100+) in all three species is consistent with pollinator limitation (Burd *et al.* 2009; Burns *et al.* 2019).

In Fabaceae, 84 % of species had *P:O* ratios between 30 and 189, values in the facultative autogamy range (Cruden 1977). Floral morphology of most legume species includes a keel that fully covers the stigma and anthers, and this special structure favours self-pollination (Agbagwa and Obute 2007) and the evolution of cleistogamy. In the field, we saw that the keel and wings completely enclosed reproductive organs in most species, and handling the flowers while anthers were dehiscing usually caused pollen grains to fall on the stigma or keels. Only *Senna obtusifolia* could be considered fully autogamous (*P:O* ratio of 20), and only *Derris trifoliata* (an indigenous species) had a *P:O* ratio (557) that even approaches facultative outcrossing. In Malvaceae, four of six species had *P:O* ratios consistent with autogamy, and the other two with facultative autogamy (both self-compatible).

A general pattern was for species to have many ovules and few pollen grains per flower. Animal-pollinated and wind-outcrossed flowers usually have on the order of tens of thousands of pollen grains per flower, whereas the maximum on Pohnpei was <3500. These general results point to the importance of investing in many ovules for rare cross-pollination events (Burd *et al.* 2009), while at the same time, the chronic pollinator limitation relaxes selection on pollen number. Thus, low *P:O* ratios here are likely a consequence of both pollinator uncertainty and long-term reproduction by self-fertilization. We did find evidence that pollen number evolution is constrained by a trade-off with pollen size. The high dependence on self-fertilization and the pollen size/number trade-off suggests that the high phylogenetic signal and lack of significant differences between ancient and recent colonizers for pollen and ovule traits is due to a lack of opportunity to evolve highly outcrossed mating systems in these species.

### Self-compatibility, autofertility and pollen limitation of Pohnpei species

Hand-pollinations showed that all 11 study species were self-compatible, and 10 produced seed by autonomous selfing. Based on the high autogamy and autofertility indices, and the low *P:O* ratios and self-incompatibility indices, we concluded that nine species had facultatively or generally autogamous breeding systems and the other two were generally mixed-mating (Table 5). No species had all indicators point to outcrossing. For instance, *Crotalaria pallida* set more seeds with hand-self- than with open-pollination and so is pollen-limited by both geitonogamy and outcrossing, yet its *P:O* ratio of 87 and high *OCI* suggests that it retains some investment in outcross or geitonogamous pollination traits.

Of the four indigenous species, *Dendrolobium umbellatum* and *Vigna marina* had relatively high OCI and *P:O* ratios, suggesting some degree of outcrossing, yet even these had ISI, AI and AF values more consistent with autogamy. Both were also pollen-limited. In 6 of 11 species, open-pollinated flowers produced higher seed yields than hand outcrosses (Table 4), which could be due to geitonogamy or higher self- than outcross pollen viability. Flowers were emasculated in both open and outcross treatments, so differential handling effects should not affect this result. However, outcross pollen might have experienced greater viability loss during transfer of anthers (plants were within 500–1500 m of each other), relative to open-pollination, or to self-pollinations, in which anthers were taken from the same flower (further confirmation of incomplete dichogamy).

## Conclusions

Plant communities on small, isolated oceanic islands represent a best-case scenario for the general predictions of Baker’s law—that founders are few to one, mate and pollinator limitation is extreme and therefore that the establishment filter strongly selects against colonists with obligate outcrossing mechanisms. Baker (1967) indicated that a finding of a ‘high proportion’ of enforced outcrossing among recently established island species would be inconsistent with Baker’s law. Among 19 species considered to have become naturalized on Pohnpei in human historical times, we found no evidence of regular outcrossing in any. Furthermore, if Baker’s law is true, most outcrossing species on islands will have evolved from self-compatible founders (Baker 1967), via changes in floral function that favour outcrossing or by transitions to obligate outcrossing by dioecy, but not to self-incompatibility (since *de novo* origin of SI is exceptionally rare). Our sample of nine indigenous species did not differ in breeding system traits from their naturalized relatives. There was a universal ability to self-fertilize coupled with low *P:O* ratios, but also a diversity of individual pollinator attraction mechanisms. Thus, for the families we studied, our data are consistent with both Baker’s law and its 1967 corollary—early establishment involved self-fertilized mating patterns, whereas long-term persistence involved retention of the ability to self and a mosaic of new and old floral mechanisms that incompletely promote pollinator attraction and cross-pollination.

## Supplementary Material

plab038_suppl_Supplementary_Materials

## Data Availability

All data and scripts for analyses are available as Supplementary Files.

## Notes

**Populations & Communities. Chief Editor:** Jean Burns
