# Supplementary material for: Breeding systems of naturalized versus indigenous species provide support for Baker’s law on Pohnpei island
Source: AoB Plants. 2021 Jun 22;13(4):plab038. doi: 10.1093/aobpla/plab038 (PMC8317631; doi:10.1093/aobpla/plab038)
Supplement: plab038_suppl_Supplementary_Materials [file plab038_suppl_supplementary_materials.zip › plab038_suppl_All_Output_File 1.docx]

###PGLS Logged Pollen ovule ratio, Pollen size, and pollen number

library(ape)

library(geiger)

library(nlme)

library(phytools)

library(AICcmodavg)

dat<- read.csv("POPSL.csv", row.names = 1)

tree<-read.tree("POPS.tre")

dat1<-read.csv("Ovule.csv", row.names = 1)

dat2<-read.csv("OCI_PL.csv", row.names = 1)

nativity<-dat2$Nativity

Nativity<-dat$Nativity

PL<-dat2$loggedPL

Sp<-dat2$Species

nat<-dat1$Nativity

Specie<-rownames(dat1)

Species<-rownames(dat)

pollen.number<-dat$LPN

ps<-dat$PS

ovule<-dat1$OL

LPO<-dat$PO

oruwan<-corBrownian(phy = tree,form = ~Species)

############PGLS - GLS#####################

######Petal length and nativity status - Pagel's lambda

pl<-gls(PL~nativity, correlation = corPagel(1, phy = tree, form = ~Species, fixed = FALSE))

summary(pl)

#####Ovule and nativity status

pagel.ovule<-gls(ovule ~ nat, correlation = corPagel(1, phy = tree, form = ~Specie, fixed = FALSE) )

###ovule and nativity no phylogeny

nophy.ovule<-gls(ovule ~ nat)

###Logged pollen ovule ratio as a factor of nativity status - Pagel's lambda

pagel.po<-gls(LPO ~ Nativity, correlation = corPagel(1, phy = tree, form = ~Species, fixed = FALSE))

summary(pagel.po)

###Pollen ovule ratio as a factor of nativity - Brownian - Maximul likelihood

model.po.2<-gls(LPO ~ Nativity, correlation = oruwan, method = "ML")

summary(model.po.2)

###Pollen ovule ratio as a factor of nativity - No phylogeny

po.no.phy<-gls(LPO ~ Nativity)

summary(po.no.phy)

###Pollen ovule ratio as a factor of nativity - No phylogeny maximum likelihood

po.no.phy.2<-gls(LPO ~ Nativity, method="ML")

###Logged pollen size as a factor of nativity status - Brownian

brown.ps<-gls(ps ~ Nativity, correlation = oruwan)

summary(brown.ps)

###Logged pollen size as a factor of nativity status - No phylogeny

nophy.ps<-gls(ps ~ Nativity)

summary(nophy.ps)

###Pollen size and nativity status - Pagel's lambda

psnativity<-gls(ps~Nativity,correlation = corPagel(1, phy = tree, form = ~Species, fixed = FALSE))

summary(psnativity)

####Pollen size ~ Pollen number - Pagel's lambda

psp<-gls(ps~pollen.number, correlation = corPagel(1, phy = tree, form = ~Species, fixed = FALSE))

summary(psp)

####Pollen size ~ Pollen number - Brownian

brown.pspn.nativity<-gls(pollen.number*ps ~ Nativity, correlation = oruwan)

summary(brown.pspn.nativity)

#####Pollen number and pollen size as function of nativity status without phylogeny

nophy.pspn.nativity<-gls(pollen.number*ps ~ Nativity)

####Pollen number and nativity status

pollen.number.nativity<-gls(pollen.number~Nativity,correlation = corPagel(1, phy = tree, form = ~Species, fixed = FALSE))

summary(pollen.number.nativity)

###Pollen number as a factor of nativity status - Brownian

brown.pn<-gls(pollen.number ~ Nativity, correlation = oruwan)

summary(brown.pn)

###Pollen number as a factor of nativity status - No phylogeny

nophy.pn<-gls(pollen.number ~ Nativity)

summary(nophy.pn)
